# Evaluating the control of HPAIV H5N1 in Vietnam: virus transmission within infected flocks reported before and after vaccination

**DOI:** 10.1186/1746-6148-6-31

**Published:** 2010-06-05

**Authors:** Ricardo J Soares Magalhães, Dirk U Pfeiffer, Joachim Otte

**Affiliations:** 1 The Royal Veterinary College, Department of Veterinary Clinical Sciences, Veterinary Epidemiology and Public Health Group, London, UK, Hawkshead Lane AL9 7TA, UK; 2 School of Population Health, University of Queensland, Edith Cavell Building, Herston Road, Herston QLD 4006, Australia; 3 Food and Agriculture Organization of the United Nation, Pro-Poor Livestock Initiative, Vialle Del Terme de Caracalla, 00153 Rome, Italy

## Abstract

**Background:**

Currently, the highly pathogenic avian influenza virus (HPAIV) of the subtype H5N1 is believed to have reached an endemic cycle in Vietnam. We used routine surveillance data on HPAIV H5N1 poultry outbreaks in Vietnam to estimate and compare the within-flock reproductive number of infection (*R*_*0*_) for periods before (second epidemic wave, 2004-5; depopulation-based disease control) and during (fourth epidemic wave, beginning 2007; vaccination-based disease control) vaccination.

**Results:**

Our results show that infected premises (IPs) in the initial (exponential) phases of outbreak periods have the highest *R*_*0 *_estimates. The IPs reported during the outbreak period when depopulation-based disease control was implemented had higher *R*_*0 *_estimates than IPs reported during the outbreak period when vaccination-based disease control was used. In the latter period, in some flocks of a defined size and species composition, within-flock transmission estimates were not significantly below the threshold for transmission (*R*_*0 *_< 1).

**Conclusions:**

Our results indicate that the current control policy based on depopulation plus vaccination has protected the majority of poultry flocks against infection. However, in some flocks the determinants associated with suboptimal protection need to be further investigated as these may explain the current pattern of infection in animal and human populations.

## Background

The incidence of HPAIV H5N1 cases in poultry and humans has been particularly high in south-east Asian countries and Egypt [1]. The current pattern of poultry outbreaks in these regions suggests the presence of a reservoir of residual infection [2,3].

From late 2003 to 2006 in Vietnam, the incidence of outbreaks in poultry was particularly high in regions of the Red and Mekong river deltas. Outbreaks were associated with increased movement of poultry around the annual traditional festivities held just before and during February (the "Tet" holiday) [4]. However, since 2006, this pattern has discontinued. Across the country, HPAIV H5N1 infection primarily affected small-scale commercial premises rearing chicken or ducks in subsistence flocks [5,6]. These market-oriented flocks account for about two-thirds of poultry production and half of direct marketing in Vietnam [7,8]. In addition to poultry cases, Vietnam has the second highest number of HPAIV H5N1-related human fatalities in the world [9]. Between 2003 and May 2010, there were 119 confirmed HPAIV H5N1 cases and 59 deaths in humans, representing a case-fatality rate of 49.6 percent.

In the early stages of the epidemic, the institutional responses in affected areas throughout Vietnam were administratively and temporally inconsistent. Measures included various large-scale depopulation (stamping-out) policies, and restrictions on poultry movement, breeding of certain poultry species, and the sale of live poultry in wet markets [10]. In September 2005, the Department of Animal Health of Vietnam started HPAIV H5N1 vaccination campaigns of susceptible poultry flocks. These campaigns were heterogeneous in terms of the timing of spatial coverage, the vaccine types used and the poultry species targeted [11].

An assessment of the efficacy of disease control measures is essential for guiding future policy. The reproductive number of infection, *R*_0_, is an averaged epidemiological property of a randomly mixing population with complete susceptibility to the infectious agent and represents the number of secondary infectious cases produced by a typical infectious case during its entire life expectancy [12]. The *R*_*0 *_measures the transmissibility of an infectious agent in a given host population and thus is a sensitive indicator of the impact of disease control interventions applied at flock and herd level [13]. The estimation and evaluation of within-flock transmissibility of avian influenza viruses is important, as this influences the flock-to-flock transmissibility and enables the development of predictive models of large-scale disease control interventions [14-17].

Few studies have assessed the efficacy of disease control efforts in the face of HPAIV H5N1 outbreaks by estimating the within-flock *R*_0 _[18]. Instead, either within-flock transmission in industrial-type IPs or in those not subject to vaccination has been quantified. Our study aims to estimate the transmissibility of HPAIV H5N1 in a sample of affected flocks in Vietnam. In doing so, we compared estimates for outbreak periods before and during the systematic vaccination campaigns, for different phases within each of the outbreak waves, and for flocks differing in size and species composition.

## Results

### HPAIV H5N1-infected IPs

**Figure 1:**
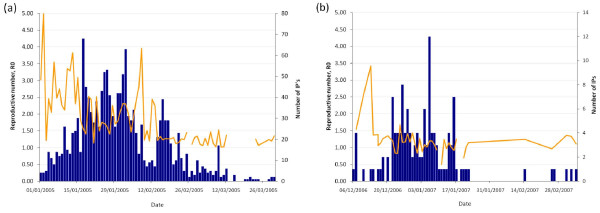
**Temporal progression of the newly reported infected premises (IPs) (blue bars) and 3-day moving average of the daily within-flock reproductive number *R*_0 _(yellow line), estimated for (a) 924 of IPs from Period I (from 1^st ^January of 2005 to 29^th ^March 2005) and for (b) 106 IPs from Period II (from 6^th ^December 2006 to 7^th ^March 2007) in Vietnam**.

Figure 1 shows the time course of the number of IPs for the two outbreak periods considered in the analysis. Using log-transformed daily incidence of reported-infected flocks we found for Period I that the exponential growth of the epidemic had reached its peak on 17 January 2005 (data not shown). One week later incidence reached a second peak with duration of 13 days.

A summary of IP characteristics regarding population sizes and observed mortality during both outbreak periods is presented in Table 1. During the two outbreak periods most IPs were duck flocks containing less than 1,500 head of poultry (88% and 92% respectively) located in the southern part of the country. The proportion of IPs with less than 50 head of poultry (i.e. backyard IPs) was 9% (93/1005) for Period I and 30% (34/114) for Period II. In addition, most mixed poultry species IPs (i.e. 92% for Period I and all in Period II) had less than 1,000 head.

**Table 1:** Number of reported HPAIV H5N1 infected premises (IPs) by flock size and flock mortality categories for Period I (i.e. pre-vaccination period) and Period II (i.e. post-vaccination period) outbreak periods.

	Pre vaccination IPs				Post vaccination IPs			
	**Chicken**	**Duck**	**Mixed**	**Other**	**Chicken**	**Duck**	**Mixed**	**Other**

**Total number(proportion)**	318(0.32)	577 (0.57)	72 (0.07)	38(0.04)	13(0.11)	91(0.8)	4(0.04)	6(0.05)

**No. of birds initially at risk(proportion)**								

*1-50 head*	58(0.18)	27(0.05)	8 (0.11)	0	7(0.54)	18(0.2)	3(0.75)	6(1)

*51-200 head*	77(0.24)	96(0.17)	25 (0.35)	1(0.03)	2(0.15)	23(0.25)	0	0

*201-500 head*	74(0.23)	152(0.26)	22 (0.31)	0	0	18(0.2)	1(0.25)	0

*501-1,000 head*	46(0.14)	154(0.27)	11 (0.15)	0	1(0.08)	18(0.2)	0	0

*1,001-1,500 head*	21(0.07)	76 (0.13)	1 (0.01)	1(0.03)	2(0.15)	7(0.08)	0	0

*1,501-3,000 head*	21(0.07)	55 (0.10)	1 (0.01)	6(0.16)	0	6(0.07)	0	0

*3,001-6,000 head*	15(0.05)	13 (0.02)	2 (0.03)	10(0.26)	0	1(0.01)	0	0

*>6,000 heads*	6(0.02)	4 (0.01)	2 (0.03)	20(0.53)	1(0.08)	0	0	0

**No. of birds reported dead (proportion)**								

*1-20 head*	58(0.18)	37(0.06)	9(0.13)	0	9(0.69)	25(0.27)	4(1)	6(1)

*21-50 head*	67(0.21)	82(0.14)	20(0.28)	1(0.03)	1(0.08)	21(0.23)	0	0

*51-150 head*	71(0.22)	145(0.25)	24(0.33)	0	0	16(0.18)	0	0

*151-300 head*	50(0.16)	123(0.21)	6(0.08)	10(0.26)	0	12(0.13)	0	0

*301-500 head*	36(0.11)	87(0.15)	5(0.07)	4(0.11)	1(0.08)	6(0.07)	0	0

*501-1,000 head*	18(0.06)	63(0.11)	5(0.07)	5(0.13)	1(0.08)	7(0.08)	0	0

*>1,001 head*	18(0.06)	40(0.07)	3(0.04)	18(0.47)	1(0.08)	4(0.04)	0	0

Across both waves, within-flock mortality was found to be significantly higher in chicken IPs with more than 1,000 head, in duck IPs with more than 500 head and in mixed poultry species IPs with more than 3,000 head (*P *< 0.05) than in chicken IPs less than 50 head. There was a marginally significant reduction in within-flock mortality in Period II compared with Period I (*P *= 0.057).

### Estimates of within-flock virus transmission

Figure 1 shows the temporal progression of the estimates of within-flock *R*_*0 *_for the outbreak periods considered in the analysis. Our results indicated that, for Period I, IPs in the initial 17 days of the epidemic (1st of January to the 17th of January; exponential growth) had above average within-flock transmission estimates (Figure 1a). During that period, chicken IPs of farm sizes larger than 1,500 head and duck IPs of less than 50 head had higher within-flock *R*_0 _than chicken IPs of less than 50 head of poultry. In addition, our analysis suggested a second peak of within-flock transmission occurred in the first week of February. A similar temporal pattern of within-flock *R*_0 _was found during Period II (Figure 1b), where IPs reported at the beginning of the epidemic had higher within-flock transmission estimates than subsequent IPs. The highest within-flock *R*_0 _in the early stages of Period II (also during the exponential growth phase of the epidemic) was estimated in duck IPs of 200 to 1,500 head of poultry followed less than a week later by chicken IPs of less than 50 head of poultry. Across the remainder of Period II, the within-flock *R*_0 _of IPs of different poultry species and size composition did not differ significantly and was significantly above unity (*P *< 0.05).

The distributions of the within-flock *R*_0 _estimates for both outbreak periods examined are summarized in Table 2. Our results for Period I showed that mean within-flock *R*_0 _did not significantly differ between IPs of different species composition (*P *> 0.05). During Period II, the mean within-flock *R*_0 _was estimated to be significantly higher in duck IPs of flock sizes between 50 and 200 and between 500 and 1,500 head of poultry compared to duck flocks of sizes less than 50 head (*P *< 0.05). Comparisons between outbreak periods indicated that the mean within-flock *R*_0 _was significantly lower during Period II when compared to Period I (95%CI: -1.16, -0.48; *P *< 0.05). There was no such difference between both outbreak periods for mixed poultry species IPs and for duck IPs with flock sizes above 500 head of poultry (*P *> 0.05). However, the mean estimate of within-flock *R*_0 _for Period II was still significantly above the threshold of transmission (*R*_0 _> 1) (*P *< 0.001). The estimated within-flock *R*_0 _in duck IPs less than 50 head (*R*_0 _= 0.96) for Period II was not significantly below the threshold of transmission (*P *= 0.635).

**Table 2:** Within-flock reproductive ratio (*R*_0_) estimated in 924 IPs reported during Period I (i.e. pre-vaccination period) and in 106 IPs reported during Period II (i.e. vaccination period).

Infected Premise Species/Size	**Reproductive number, *R***_***0***_** (Mean; 95%CI; N)**	
	**Pre-vaccination IPs**	**Post-vaccination IPs**

**All IP's**	1.99; 1.87, 2.10; 924	1.17; 1.09 - 1.25; 106

**Chicken flocks only**		

*Pooled*	2.16; 1.94, 2.39; 297	1.07; 0.90, 1.23; 12

*1-50 heads*	2.03; 1.71, 2.36; 58	1.04; 0.76, 1.32; 7

*51-200 heads*	1.90; 1.55, 2.25; 77	0.80; 0.68, 1.11; 2

*201-500 heads*	2.28; 1.8, 2.74; 74	NA

*501-1,000 heads*	1.92; 1.33, 2.50; 46	1.02; 0.87, 1.24; 1

*1,001-1,500 heads*	2.77; 1.45, 4.09; 21	1.34;1.03, 1.71; 2

*>1,500 heads*	2.86; 1.40, 4.31; 21	NA

**Duck flocks only**		

*Pooled*	1.96; 1.81, 2.11; 560	1.21; 1.12, 1.31; 90

*1-50 heads*	2.02; 1.53, 2.50; 27	0.96; 0.72, 1.20; 18

*51-200 heads*	1.85; 1.55, 2.14; 96	1.36; 1.12, 1.59; 23

*201-500 heads*	2.16; 1.84, 2.48; 152	1.09; 0.95, 1.24; 18

*501-1,000 heads*	1.90; 1.61, 2.19; 154	1.27; 1.12, 1.41; 18

*1,001-1,500 heads*	1.92; 1.48, 2.36; 76	1.46; 1.02, 1.90; 7

*>1,500 heads*	1.55; 1.18, 1.92; 55	1.31; 1.01, 1.61; 6

**Mixed poultry species**		

*Pooled*	1.91; 1.53, 2.29; 67	1.01; 0.74, 1.23; 4

*1-50 heads*	3.20; 2.19, 4.21; 8	1.04; 0.83, 1.25; 3

*51-200 heads*	1.61; 1.24, 1.97; 25	NA

*201-500 heads*	1.28; 1.10, 1.45; 22	0.92; 0.74, 1.31; 1

*501-1,000 heads*	2.45; 0.86, 4.04; 11	NA

*1,001-1,500 heads*	1.47; 1.17, 1.85; 1	NA

*>1,500 heads*	NA	NA

### Univariable analysis

We investigated the strength of association between the outcome variables a) within-flock *R*_0 _on a continuous scale (Model 1) and b) within-flock *R*_0 _estimates categorised as lower than unity or not (Model 2) and three flock-level risk factors (i.e. "flock species", "flock size" and "epidemic wave"). In the univariable analyses all variables reached the decision criterion for variable selection except "flock species" for Model 2 (Table 3).

**Table 3:** Univariable results: within-flock *R*_*0 *_vs. flock attributes.

Variable	Model 1		Model 2	
	**Coefficient (95%CI)**	***P***	**OR (95%CI)**	***P***

**Flock species ***(Ref: Duck flocks)*				

*Mixed poultry species*	0.006 (-0.416,0.428)	0.977	2.054 (0.957,4.411)	0.065

*Chicken flocks*	0.264 (0.030,0.498)	0.027	1.580 (0.972,2.570)	0.065

**Flock Size***(Ref: <50 head)*				

*>50-500 head*	0.107 (-0.242,0.456)	0.549	0.165 (0.096,0.286)	<0.001

*>500-1,500 head*	0.111 (-0.254,0.476)	0.551	0.109 (0.055,0.216)	<0.001

*>1,500 head*	0.064 (-0.351,0.479)	0.762	0.160 (0.073,0.348)	<0.001

**Epidemic wave***(Ref: Period I)*				

*Period II*	-0.821(-1.163,-0.478)	<0.001	4.504 (2.689,7.544)	<0.001

OR: Odds Ratio; SE: Standard Error; CI: Confidence Interval

### Multivariable analysis

In Model 1, only the variable "epidemic wave" retained significance at a *P *< 0.05. Our results suggest that the within-flock *R*_0 _estimates for Period II are significantly lower compared with Period I. The coefficient of determination of the final model Model 1 was R^2 ^= 0.41. In Model 2, the variables "flock size" and "epidemic wave" retained significance at a *P *< 0.05 (Table 4). Our results suggest that after controlling for the effect of flock size, IPs of Period II had increased odds of having within-flock *R*_0 _below unity compared to IPs of Period I. The goodness-of-fit of the final multivariable model to the data, as assessed by the Hosmer-Lemeshow goodness of fit test, was adequate (*P *= 0.816).

**Table 4:** Multivariable results for Model 2: within-flock *R*_*0 *_being below unity vs. flock attributes.

Variable	OR (95%CI)	*P*
**Flock Size ***(Ref: <50 head)*		

*>50-500 head*	0.202 (0.115,0.355)	<0.001

*>500-1,500 head*	0.134 (0.067,0.269)	<0.001

*>1,500 head*	0.209 (0.094,0.464)	<0.001

**Epidemic wave ***(Ref: Period I)*		

*Period II*	3.114 (1.785,5.432)	<0.001

OR: Odds Ratio; SE: Standard Error; CI: Confidence Interval

## Discussion

This study compared and characterized the transmissibility of HPAIV H5N1 in affected flocks in Vietnam, before and after the introduction of a control policy that included vaccination. Our results show that measuring within-flock virus transmissibility can improve understanding of the epidemiology and effectiveness of control strategies against HPAIV H5N1 infection in endemic countries.

Control policies based on flock depopulation (such as in Period I) are expected to have a direct impact on the effective within-flock contact rate while control involving depopulation plus preventive vaccination of flocks (such as in Period II) will contribute with the added impact on the properties of the natural history of infection. However, factors such as delays in disease reporting and operational constraints of stamping-out interventions will influence the effectiveness of both control strategies. This circumstance allows the generation of chains of infection within a flock that would not have happened if birds had been stamped-out at an earlier stage. In contrast, vaccination for HPAIV H5N1 is expected to have an individual (direct) effect (e.g. increase the incubation period, reduce the number of infectious virus excreted as well as reduce the duration of infectiousness) and a population (indirect) effect (e.g. via herd immunity) on the natural history of infection [19]. Any partially immune individual introduced infectious into an unvaccinated flock will shed less HPAIV H5N1 particles and be infectious for a shorter period [20,21]. Similarly, the population effect of a vaccination programme that reaches flocks in the vicinity or those that are part of the trade relationships of an unvaccinated flock will gradually contribute to herd immunity (despite the fact that some transmissions may still occur) [22]. In addition, vaccination is expected to contribute to a reduced environmental contamination.

Our study findings suggest a differential effect of disease control interventions on within-flock virus transmissibility. Inspection of the temporal progression of estimates of within-flock *R*_0 _indicates that IPs of different flock size and species composition are likely to have had different roles in the transmission dynamics during different phases of the outbreak periods examined in our study. Our results show that the within-flock *R*_0 _of flocks with similar species and size composition varied over the course of each epidemic wave, and higher estimates were found in earlier phases of each epidemic wave. Based on the assumption of constant contact structure and transmission dynamics among flocks of similar species and size composition, this variation may be due to the effect of disease control interventions. The highest within-flock *R*_0 _observed in the initial phases of the outbreak periods may be attributed to the fact that flocks with high effective contact rates and those that experienced delayed application of control interventions (either by delayed disease notification or operational delays) were disproportionally represented. Our results indicate that chicken IPs with flock size larger than 1,500 head had the highest *R*_0 _estimates during the initial (exponential) phase of Period I. Previous studies have shown that HPAIV H5N1 spreads more rapidly both in farms with larger numbers of chicken houses in use and among layer chickens (compared with broilers) [23]. Similarly, IPs with flock sizes less than 50 head, and duck flocks of 200 to 1,500 head were also identified to have the highest within-flock *R*_0 _estimates during the initial phases of Period II. Consequently, it is likely that these flocks could have contributed to localised spread of infection observed during Period II - they are characterised by a free-ranging husbandry system and mortality often remains unnoticed and/or unreported mainly due to the absence of records and difficulties in monitoring [6]. In addition, it has been suggested that the quality of the depopulation policy between and within each of the outbreak periods was inconsistent (see section#3 in Additional file 1). Therefore, it is also likely that IPs in the initial phases of the outbreak periods - due to operational constraints resulting from having to deal with a large number of outbreaks simultaneously - had experienced delays in receiving disease control interventions compared to later IPs. As a result, both high contact rates and delayed application of interventions provided an opportunity during the initial phases of the outbreak periods for infectious birds to generate more and longer infection chains within flocks. This has important implications with respect to outbreak containment as it could result in large numbers of infected birds being present within such flocks prior to reporting - if these flocks are involved in trade before movement restrictions is enforced, they can make a significant contribution to geographical spread of the virus.

Our results suggest that the estimates of within-flock *R*_0 _for Period I are broadly comparable to those reported for HPAIV H5N1 infected chicken flocks from Thailand (e.g. 2.26-2.64, for infectious periods of 1 and 4 days respectively) [18]. Any observed differences might be due to differences in the quality of the control policies applied, or the population demographics and husbandry of flocks in each country. These factors are known to affect the underlying contact structure of a population which is an important driver of virus transmission [24]. In addition, the quality of the mortality data and the estimation methods used in the current study could have contributed to estimation bias and consequently to differences in *R*_0 _estimates between both studies.

The findings of our study are also consistent with avian influenza vaccine trials that suggest that the use of vaccines is expected to reduce virus transmission and associated mortality among poultry by increasing the incubation period and reducing virus shedding [20,21]. Our results indicate that IPs reported during Period II develop lower mortality than their pre-vaccination counterparts. However, because farmers often use mortality as a flock health indicator this may contribute to the reduced ability of farmers and animal health workers to recognise the presence of disease. Other studies in unvaccinated populations have found that farmers recognize the abnormally high mortality resulting from HPAIV H5N1 approximately 5 days after infection [23].

Overall, our results showed that the mean within-flock *R*_0 _of IPs from Period II was significantly lower compared to Period I - however, this reduction was not significantly below the threshold of transmission. Considering that IPs from Period II were classified as unvaccinated, this finding suggests that suboptimal vaccine coverage will lead to the re-emergence of outbreaks. Apart from issues related to the quality of protection provided by the vaccine, the overall effectiveness of the vaccination campaigns in target species is expected to be undermined by factors that deter farmers with commercial sized flocks from presenting their flocks for vaccination and operational issues for vaccine delivery. The former may be linked to the length of vaccine-withholding period and rumors concerning adverse reactions to the vaccine while the latter may be affected by issues such as the training and payment of vaccinators, the breakdown or spoilage of vaccine stocks and the fact that poultry husbandry is associated with rapid turn-over of at-risk populations. These factors have previously been documented in Vietnam and are likely to act as constraints to suboptimal vaccine coverage and consequently the effectiveness of the current vaccination campaigns [25]. These concerns are supported by the results of our multivariable analysis which indicate that commercial sized flocks (more than 50 head) have reduced odds of having within-flock *R*_0 _estimates below unity. Therefore, in Vietnam, a control strategy based on nationwide HPAIV H5N1 vaccination campaigns should take into account the heterogeneities mentioned above [26]. This is particularly imperative when field evidence from other countries indicates that vaccination alone will not achieve disease elimination unless it is managed appropriately as part of a wider disease control strategy [27-31].

Our results should be interpreted considering the study's assumptions and limitations. Firstly, in our analyses, we assume that within-flock virus transmission will be halted in a scenario of total flock depopulation. However, if these flocks were left to develop further chains of infection (due to delays in disease reporting and depopulation) the within-flock *R*_0 _estimate would be different. Secondly, our estimation method assumes a single introduction as the source of infection for an IP. It is known that pre-emptively culled farms were allowed to repopulate their flocks after a period of 60 days, but data on the proportion of farms which repopulated and the infection status of birds involved are unreliable. Similarly, there is no information with respect to the previous history of infection of these flocks and about the source leading to the current infection. Thirdly, we assume that the contact structure of flocks of similar species and size composition is constant, which in some situations may not be the case due to farm-to-farm variation in husbandry systems. Fourthly, we have assumed that all flocks included in the analysis were closed populations which, in the case of free-ranging flocks, may have led to an overestimation of the *R*_0 _- however, the available data do not allow us to differentiate whether flocks included in the analysis were scavenging or not at the moment when they were reported infected.

The results of this study indicate that vaccination has protected the majority of poultry flocks against infection. However, our findings also provide evidence of the potential shortcomings of the current vaccine-based policy. Despite the control programme protecting the majority of farms as reflected in the very much reduced national incidence, the policy was not as effective in the flocks included in our analyses. To prevent similar cases, which are likely to become a continuing source of infection for poultry and humans, it is important to understand the factors behind the failure of the control policy in such flocks. In addition, we anticipate that if vaccination continues to be included as part of a sustainable disease control programme, efforts should be focussed on training farmers in disease prevention in addition to disease recognition, as the latter is likely to be compromised in a vaccinated population. Efforts must also be made to reduce operational delays in the implementation of disease control interventions after the recognition of the initial IP.

## Conclusions

The results of this study show marked differences in the within-flock transmissibility of HPAIV H5N1 in Vietnam, before and after the introduction of a control policy that included vaccination. Our study showed:

• Within-flock mortality was lower in IPs reported after the introduction of a control policy that included vaccination compared to pre-vaccination IPs.

• Higher within-flock *R*_0 _estimates were found in earlier phases of the outbreak periods examined.

• The mean within-flock *R*_0 _in reported IPs was significantly lower in Period II (control based on depopulation plus vaccination) compared with Period I (control based on depopulation), but this reduction was not significantly below unity.

• After controlling for the effect of epidemic wave, commercial flocks (>50 head) had reduced odds of having within-flock *R*_0 _estimates below unity compared to flocks of smaller size (<50 head).

Given the above, the observed effect is likely to be associated with the type and quality of disease control operations that were implemented during particular periods of the epidemic waves.

## Methods

### Outbreaks of HPAIV H5N1 in poultry

We analysed outbreak surveillance data on known infected flocks in Vietnam from two outbreak periods: one outbreak period from 1^st ^December 2005 to 29^th ^March 2005 (i.e. before the first systematic vaccination campaign - Period I) and another outbreak period from 16^th ^November 2006 to 7^th ^March 2007 (i.e. during the third and fourth nationwide systematic vaccination campaigns - Period II) (see section #2 in Additional file 1). These periods reflect the second and fourth outbreak waves that occurred in the country since 2003/7 [4].The datasets used in the analyses only contained known affected flocks and were provided by the Epidemiology Division of the Department of Animal Health of the Ministry of Agriculture and Rural Development, Hanoi, Vietnam. All flocks included in the study were officially confirmed positive for HPAIV H5N1 virus by virus isolation and PCR at the National Centre of Veterinary Diagnostics (NCVD). In addition, the outbreak investigations conducted in relation to all flocks from Period II included in this analysis revealed that they had not been vaccinated [1].

### Estimation of the within-flock reproductive number, *R*_0_

We estimated *R*_0 _based on the general state-transition epidemic model adapted to the known properties of HPAIV H5N1 infection within a poultry flock. We applied the theory of moments of martingales to a general epidemic model informed by surveillance data from each infected flock included in the dataset (see section #3 in Additional file 1). The method was first described by Becker et al [32] and applied to infectious disease quantification studies in humans and animal populations [33-35]. It has been described as a robust method for statistical estimation of the infection potential of a population when there is incomplete follow up of its infectious status [36,37]. The model formulation assumes a) uniform within-flock mixing, b) flocks homogeneity, and c) that an outbreak within an IP is a result of a single introduction. Finally, we estimated the mean within-flock *R*_0 _for predefined species and size categories (see section #2 in Additional file 1). The estimation procedures were implemented in the statistical software R [38].

### Standard statistical methods

Comparisons between the mean within-flock *R*_0 _in subsets of data were conducted using *t*-tests whenever data fulfilled the assumptions of normality and equal variances. Otherwise a non-parametric Kruskal-Wallis test was applied. One sample *t*-tests were performed to assess if the group mean within-flock *R*_0 _was different from unity.

The statistical analysis leading to the identification of factors associated with within-flock *R*_0 _estimates was carried out in two phases using 1) within-flock *R*_0 _in a continuous scale outcome (Model 1) and 2) within-flock *R*_0 _categorised into a threshold value (i.e. *R*_0 _< 1 or *R*_0 _≥ 1) (Model 2). Firstly, flock species (categorized into duck flocks, mixed poultry species and chicken flocks), flock size (categorised into <50 head, 50-500 head, 500-1,500 head and >1,500 head) and epidemic wave (categorised into Period I and Period II) were screened using univariable linear regression (Model 1) and logistic regression (Model 2) based on a liberal *P*-value of 0.20 in the likelihood-ratio test. Secondly, all factors significant in the screening phase were considered for inclusion through a manual backward stepwise variable selection process in a multivariable linear regression (Model 1) and in a logistic regression (Model 2) analysis. The criterion for removal of risk factors was based on the likelihood ratio test with a significance level of *P *> 0.05. Biologically meaningful first-order interaction terms were also tested for statistical significance. The goodness-of-fit of the final multivariable Model 1 was assessed by inspection of the coefficient of determination (R^2^) and for the final multivariable Model 2 was assessed by the Hosmer-Lemeshow goodness-of-fit test [39].All statistical analyses were conducted using the statistical software Stata/SE Version 9.2 (Stata Corporation) [40].

## Authors' contributions

RJSM conceived the study, conducted the analysis and wrote the manuscript. DUP supervised the study and critically revised the manuscript. JO critically reviewed the manuscript. All authors read and approved the final manuscript.

## Supplementary Material

Additional file 1 **Supplementary technical information**. Click here for file
