# Supplementary material for: Evaluating the control of HPAIV H5N1 in Vietnam: virus transmission within infected flocks reported before and after vaccination
Source: BMC Vet Res. 2010 Jun 5;6:31. doi: 10.1186/1746-6148-6-31 (PMC2898779; doi:10.1186/1746-6148-6-31)
Supplement: Additional file 1 — Supplementary technical information. [file 1746-6148-6-31-S1.DOC]

## Evaluating the control of HPAI H5N1 in Vietnam: virus transmission within infected flocks reported before and after vaccination.

## Technical Appendix

# Background on HPAI H5N1 control policies in Vietnam

The two initial epidemic waves occurred during the period of December 2003 to May 2005 during which vaccination was not included as part of the national disease control program. Vaccination-based control policy interventions are officially put in place in September 2005 until this date. The infected premises (IPs) of the third HPAI H5N1 epidemic wave are reported in October 2005 until December 2005. The fourth and fifth epidemic waves occurred during November 2006 to November 2007 when countrywide vaccination-based disease containment policies were in place. During the first epidemic wave, depopulation dominated the portfolio of disease control measures and the extent of its application was not systematically documented [1]. Overall, the implementation of depopulation during this period is expected to have varied in nature, quality, time and space across the country in contrast to later epidemic waves and this would have contributed to a higher heterogeneity of within-flock transmission estimates compared with those for the second wave. In addition, at that time, the laboratory diagnostic capacity of the country was insufficient, which increased the confirmation time of suspected IPs. This in turn vindicated the application of rapid depopulation of the flocks. Finally, the technical guidelines for prevention and control of avian influenza were released 6 months after the first outbreaks of the first wave were reported. During the third outbreak wave, depopulation upon identification of an IP was applied and flocks within different provinces were already being vaccinated. Surveillance data available for this period did not allow unbiased ascertainment as to whether the IPs recorded in the dataset were in an area where vaccination was being put in place.

During the second epidemic wave (2004-2005), depopulation was implemented at the village level and poultry had not been vaccinated. IPs and pre-emptively culled farms were allowed to repopulate their flocks after a period of 60 days but data on the proportion of farms which repopulated are unreliable.

During the 2006-2007 epidemic (fourth epidemic wave) a protection zone (PZ) was established which equals the area of the village where the IP was located. Within the PZ the IP was depopulated and movement restricted. For this period, pre-emptive depopulation of flocks in the PZ was no longer compulsory as was the case during the first to third waves; poultry farms within the PZ were offered the option of compensated depopulation. By the time the first outbreak of the fourth epidemic wave was reported, three vaccination campaigns had taken place and the fourth campaign was ongoing. This campaign focused on poultry flocks located in 33 provinces, which had a history of being high-risk areas for HPAI H5N1 outbreaks in poultry. A H5N2 vaccine (Nobilis, Intervet) was made available at cost for commercial breeders of great grandparent (GGP) and grandparent (GP) stocks. This vaccine required primo-vaccination followed by a booster after a 4 week interval and a second booster another 4 months later. An inactivated Re-1 strain, reassortant H5N1 vaccine (Homologous Habin-Weike Reassortant Avian Influenza vaccine) was used for vaccination of broiler ducks, the cost of which was fully borne by the government. With this vaccine, ducks required a booster one month after primo-vaccination and a second booster 6 months thereafter. The Homologous Habin-Weike Reassortant Avian Influenza vaccine was also being used in chicken broiler flocks as a single booster vaccine (i.e. two injections). In addition a H5N1 recombinant vaccine (Trovac) had been introduced for use in chicken breeder hatcheries and applied to day-old-chicks.

# Data quality and data handling issues

Outbreak surveillance data generated during the period from December 2003 to September 2008 is publically available at the World Organization for Animal Health (OIE) which is officially notified by the Department of Animal Health of the Ministry of Agriculture and Rural Development of Vietnam (DAH-Vietnam) [2]. The HPAI H5N1 outbreak surveillance data considered for this study was kindly facilitated by the Epidemiology Division of DAH-Vietnam. Data concerning flock type (i.e. species reared), total poultry population initially at risk (i.e. flock size) and the number of observed bird deaths were available.

Upon discussion with senior animal health officials and epidemiologists at DAH-Vietnam we made the decision of excluding the data from the first (i.e. December 2003 to March 2004) and third (i.e. October 2005 to December 2005) epidemic waves from the analysis. The criteria for exclusion were based on evidence about the lack of consistency across the country in data recording for the first epidemic wave and in disease control policies for the first and third epidemic waves[1]. Furthermore, data aggregation was a caveat of the outbreak surveillance data generated during the first epidemic wave while uncertainty with respect to regional vaccination status of IPs recorded during the third epidemic wave were issues that supported the exclusion of these data.

For the comparative investigation between periods of vaccine-based control versus depopulation-based control we considered a dataset which included outbreak surveillance data from the second (i.e. depopulation-based control; Period I) and fourth (i.e. vaccination-based control; Period II) epidemic waves.

The dataset for Period I included infected premises (IPs) which were pre-emptively depopulated as part of the control policy; however, these records were excluded from the final dataset for analysis. None of the IPs of Period I had been vaccinated.

The official reporting data contained a total of 1005 IPs for the period 1^st^ January 2005 to 1^st^ May 2005 (second epidemic wave; Period I) and a total of 114 IPs for the period from 16^th^ November 2006 to 7^th^ March 2007 (fourth epidemic wave; Period II) with observed bird mortality due to H5N1 infection. During these epidemic periods a total of 545 communes from 32 (50%) provinces of Vietnam were affected and in 90% (491/545) of affected communes the number of IPs with reported mortality ranged from one to four. The majority of IPs during Period I (81%; n=818) and Period II (96%; n=110) epidemic waves were located in provinces within the Mekong River delta (in South Vietnam). For the Period II the dataset did not contain any IPs located in provinces other than the Red River (North Vietnam) and Mekong River deltas.

We were interested in obtaining unbiased estimates of the within-flock *R*_0_ therefore the final dataset for analysis needed to contain unbiased records of estimates of mortality and respective denominators for each IP. Therefore, and based on discussions with the DAH-Vietnam officials, we anticipated recording biases of the total mortality observed in some of the flocks particularly in those with flock sizes greater than 1,500 heads. This lead to the inclusion in the final dataset for analysis of 924 flocks for Period I (i.e. 8% (81/1005) were excluded) and 106 flocks for Period II (i.e. 7% (8/114) were excluded).

We could have introduced biases in our analysis for Period II (vaccination-based control) if vaccinated flocks had been included; in this scenario, mortality in vaccinated flocks would not be a good indicator of infection. According to information provided by the DAH-Vietnam and information available at OIE (at the World Animal Health Information Database (WAHID) Interface) none of the flocks considered in the analysis for this period was listed as being vaccinated at the moment when the outbreak was reported.

For the comparative investigation of the reproductive number of infection between flocks of different type/sizes compositions we have classified infected premises of the final dataset for analysis into discrete categories pertaining to their expected scale of production. The Vietnam’s poultry sector has been historically sub-divided according to different criteria [3-5] and the IP categorization used assumes 6 cut-offs based on the frequency distribution of IPs by flock type and size composition (see Table 1). This was also performed in order to take into account possible non-linearity of effects of size during statistical analysis.

Finally, we argued whether mortality in duck flocks due to H5N1 might not be a good indicator of virus transmission. Indeed, available evidence suggests that the initial 1997-2004 highly HPAI H5N1 viruses in domestic and wild waterfowl tended to behave like low-pathogenic avian influenza viruses in other avian species. In addition, experimental evidence specific to viruses isolated from Vietnam during outbreak in 2003 and 2004 show that those tended to replicate and transmit efficiently in ducks but exhibited variable pathogenic potentials in ducks, ranging from the complete absence of clinical disease (intravenous pathogenicity index [IVPI] 0.0 in ducks) to severe neurological dysfunction and death (IVPI of 3.0 in ducks) [6]. However, recent experimental evidence for viruses lineages circulating in Viet Nam from 2005 onward – which corresponds to the epidemic periods analysed in the manuscript – suggests that these were highly virulent in ducks , causing 100% mortality in 2-week old Pekin ducks within four days of virus challenge [7, 8]. In addition, mortality figures caused by these viruses are suggested to correlate well with mortality figures observed in chicken and these viruses were much more virulent to ducks compared with viruses circulating before 2005 [7]. Based on this recent evidence the decision was made to include the outbreak data from duck flocks.

# Estimation of the within-flock reproductive number of infection, *R*_0_ based on the method of moments

The basic reproductive number *R*_0_ allows the quantification of the infection potential of a population and is an averaged epidemiological property that summarises the relationship between the effective contact rate (*β*) and the mean duration of infectiousness (1/*α)* adjusted for the size of the initial population at risk. It is difficult to estimate *β* when complete follow-up data for animals are not available [9]. In order to do estimate the within-flock reproductive number, *R*_0_ we have assumed that the same category of flock size/type would have the same degree of mixing as their homologous flocks. In this scenario we estimated the within-flock *R*_0_ which will be the average number of secondary cases created by a typical infectious bird from the point it became infectious until the moment of flock depopulation.

The theory of moments of martingales applied to the general epidemic model allows the estimation of the parameters for *β* and 1/*α* simultaneously [10]. This quantitative approach takes into account the final size of an outbreak, the initial population at risk and time. We have used the expression below to calculate the infection potential of a flock up to the time point when it was culled, considering the following relationships,

$$\hat{\theta}^{f}=\frac{1}{c_{T}^{f}}\ln\left(\frac{s_{0}^{f}+0.5}{\left(N_{0}^{f}-c_{T}^{f}+1\right)-0.5}\right)$$

where *f* is the infected flock.The parameter (*θ*) to be estimated is conceptually equal to *β*/*α* which, as mentioned above, are the primary ingredients for the estimation of *R*_0_. The necessary parameters for the estimation of *θ* were the total number of birds at risk in the flock at the start of the outbreak ($s_{0}^{f}=N_{0}^{f}-1$), the number of birds in the flock at the start of the outbreak ($N_{0}^{f}$) and the cumulative number of observed deaths (new cases) when the infection chain was stopped by culling ($c_{T}^{f}$). Assuming there was a single case introduced to the flock, the actual initial population at risk at the start of the outbreak for a given flock was estimated as $N_{0}^{f}$-1, with $N_{0}^{f}$ being the recorded total population of the flock at the moment when infection was reported. Previous studies have shown that mortality observed on a given day had a positive linear association with time after initial infection and that without any intervention, entire flocks would die within 12 days of introduction of the HPAI virus to the infected farm [11]. However, the aim of our analysis was to estimate the efficacy of the control policy and not to examine the biological properties of transmission.

The *R*_0_ of an IP was calculated by multiplying *θ* by the total number of birds initially at risk in the flock. An estimator of the standard error of *θ* has also been previously described [10] and was used for the estimation of confidence intervals around the estimated *R*_0_ values.

# References
